# The complete chloroplast genome of *Bromus biebersteinii*

**DOI:** 10.1080/23802359.2021.1942263

**Published:** 2021-06-21

**Authors:** Wenxuan Du, Junfeng Yang, Yongzhen Pang

**Affiliations:** Institute of Animal Sciences, Chinese Academy of Agricultural Sciences, Beijing, China

**Keywords:** Chloroplast genome, *Bromus biebersteinii*, Gramineae

## Abstract

*Bromus biebersteinii* is a perennial gramineous grass, which is mainly distributed in Southwest Asia. In our study, we obtained the complete chloroplast genome of *B. biebersteinii* and found it is 137,189 bp in length. The GC content of its whole chloroplast genome is 38.37%. Among the 134 unique genes in the circular genome, 38 tRNA, 8 rRNA and 88 protein-coding genes were successfully annotated. We constructed the Maximum likelihood (ML) tree with 12 species, and found that *B. biebersteinii* was phylogenetically close to *Bromus vulgaris* of the genus *Bromus*.

The genus *Bromus* is one of the largest genera of the festucoid grasses (Williams et al. 2011). The plants of the genus *Bromus* are widely distributed worldwide, which includes annual, biennial and perennial plants with different ploidy levels. The plants of the genus *Bromus* have adapted to cold climates or areas that the cold season is a part of plant growth period (Sanderson et al. 2002). *Bromus biebersteinii* is a cold season grass, which showed strong drought resistance and it is a promising grass for grazing land (Safari et al. 2019). The chloroplast is an important organelle that has its own genomes, and the chloroplast genome of plants has been a focus of research in plant molecular evolution and systematics (Clegg et al. 1994). Several features of chloroplast genome have facilitated molecular evolutionary analyses. However, the chloroplast genome sequence of *B. biebersteinii* has not been reported so far. In the present study, the chloroplast genome of *B. biebersteinii* was sequenced and its gene structure features were analyzed, which is a valuable resource for further studies on the genetic evolution of Gramineae.

Seeds of *B. biebersteinii* were originally acquired from the Federal Research Center of Russia Vavilov Institute of Plant Genetic Resources (VIR) and kept at the Forage Germplasm Bank at Institute of Animal Science of the Chinese Academy of Agricultural Sciences (Beijing, E116°29′, N40°03′). The voucher specimen (FR14282) was deposited at the Herbarium of the Institute of Animal Sciences of the Chinese Academy of Agricultural Sciences, Beijing, China (http://ias.caas.cn/, Yongzhen Pang, pangyongzhen@caas.cn). After germinated in the laboratory, genomic DNA from young leaves was extracted using a DNA Extraction Kit from Tiangen Bio Tech Co., Ltd (Beijing, China). The sequencing was carried out on the Illumina Novaseq PE150 platform (Illumina Inc, San Diego), and 150 bp paired-end reads were generated. The software GetOrganelle v1.5 (Jin et al. 2018) was used to assemble the cleaned reads into a complete chloroplast genome. The chloroplast genome annotation was performed through the online program CPGAVAS2 (Shi et al. 2019) and GeSeq (Tillich et al. 2017), followed by manual correction. The assembled chloroplast genome sequence with annotation has been submitted to GenBank under the accession number MW309816.

In the present study, we found that the complete chloroplast genome of *B. biebersteinii* is 137,189 bp in length, which is a typical circular structure. The GC content of its whole chloroplast genome is 38.37%. The chloroplast genome has a total of 134 genes, including 88 protein-coding genes, 38 tRNA genes, and 8 rRNA genes. Among these 134 genes, 36 genes that encode amino acid transfer proteins (*trnK-UUU trnQ-UUG trnS-GCU trnS-UGA*, *trnG-GCC*, *trnM-CAU*, *trnS-CGA*, *trnT-GGU*, *trnE-UUC*, *trnY-GUA*, *trnD-GUC*, *trnC-GCA*, *trnR-UCU*, *trnS-GGA*, *trnT-UGU*, *trnL-UAA*, *trnF-GAA*, *trnV-UAC*, *trnM-CAU*, *trnW-CCA*, *trnP-UGG*, *trnH-GUG*, *trnM-CAU*, *trnL-CAA*, *trnV-GAC*, *trnA-UGC*, *trnR-ACG*, *trnN-GUU*, *trnL-UAG*, *trnN-GUU*, *trnR-ACG*, *trnA-UGC*, *trnV-GAC*, *trnL-CAA*, *trnM-CAU*, *trnH-GUG*), 28 genes that encode ribosomal structural proteins protein (*rps16*, *rps2*, *rps14*, *rps4*, *rpl23*, *rpl33*, *rps18*, *rpl20*, *rps12*, *rps12*, *rps11*, *rpl36*, *rps8*, *rpl14*, *rpl16*, *rps3*, *rpl22*, *rps19*, *rpl2*, *rpl23*, *rps7*, *rps15*, *rpl32*, *rps15*, *rps7*, *rpl23*, *rpl2*, *rps19*), 16 genes that encode electron transport proteins (*petN*, *ndhJ*, *ndhK*, *ndhC*, *petA*, *petG*, *petB*, *petD*, *ndhB*, *ndhH*, *ndhA*, *ndhI*, *ndhG*, *ndhD*, *ndhF*, *ndhB*), and 14 genes that encode light collection structural proteins (PSII) (*psbA*, *psbK*, *psbI*, *psbD*, *psbC*, *psbZ*, *psbM*, *psbJ*, *psbL*, *psbF*, *psbE*, *psbB*, *psbT*, *psbH*) are found in the chloroplast genome of *B. biebersteinii.*

The chloroplast genomes of 12 plant species from Gramineae and *Bambusa bambos* (KJ870988) as out-group species were downloaded from the NCBI GenBank database to inference the phylogenetic relationship of *B. biebersteinii*. The sequences were aligned using MAFFT v7 (Katoh et al. 2019). In addition, a Maximum likelihood (ML) tree based on the common protein-coding genes of 12 species by using raxmlGUI1.5b (v8.2.12) (Silvestro and Michalak 2012), and a Bayesian phylogenetic tree by using MrBayes (Huelsenbeck and Ronquist 2001), were constructed. Phylogenetic analysis with both trees shows that *B. biebersteinii* is closely related to plant species of the genera *Bromus* and *Brachypodium* (Figure 1). This study will provide important information for species identification and phylogenetic relationship in the Gramineae.

**Figure 1.**
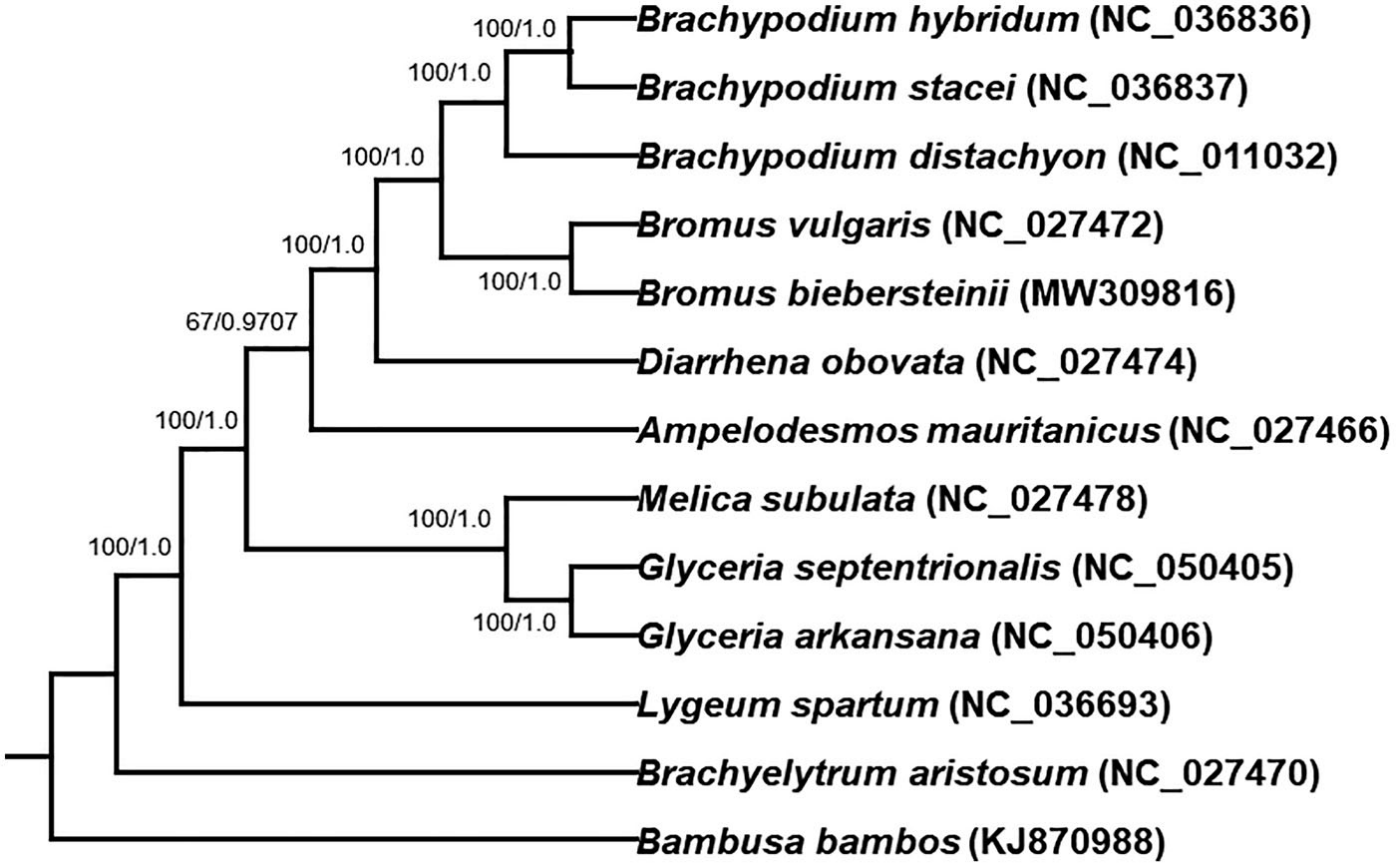
Phylogenetic tree reconstructed using maximum likelihood (ML) method based on the common protein-coding genes of 13 species of the Gramineae, with *Bambusa bambos* as the outgroup by using raxmlGUI1.5b. Numbers on the internodes refer to ML bootstrap support values/MrBayes posterior probability.

## Data Availability

The data that support the findings of this study are openly available in NCBI at Genbank with accession number MW309816. Raw sequencing data was deposited in the public repository SRA with accession number SRR13391446 (http://submit.ncbi.nlm.nih.gov/subs/sra/SUB8852316/overview)
